# AGING AGENCIES’ AND PROVIDERS’ REASONS FOR HOSTING OR NOT HOSTING GERONTOLOGY INTERNS

**DOI:** 10.1093/geroni/igac059.1971

**Published:** 2022-12-20

**Authors:** Rona Karasik, Phyllis Greenberg, Maria Kroeber, Jessica VanderWerf, Claudia Burgos Zuniga

**Affiliations:** St. Cloud State University, St. Cloud, Minnesota, United States; St. Cloud State University, St Cloud, Minnesota, United States; St. Cloud State University, St. Cloud, Minnesota, United States; St. Cloud State University, St. Cloud, Minnesota, United States; St. Cloud State University, St. Cloud, Minnesota, United States

## Abstract

Hands-on experience is essential to gerontological education and associated with numerous student benefits. Much less is known about the community agency perspective, including why they do/don’t host gerontology interns. In this national study (n=281), non-profit, for-profit, and government aging-service providers (e.g., Social Services, Health Care, Housing) were surveyed regarding their perceptions of internship benefits, challenges, and areas to improve. Unique to this study is the inclusion of both hosting (n=129) and non-hosting (n=142) agencies. Hosts’ reasons for accepting interns included: share experience/knowledge (n=81, 87%); future university relationships (n=71, 76%); intergenerational interaction for students (n=69, 74%) and clients (n=60, 65%); attract future employees (n=68, 73%); and fills unmet need at agency (n=47, 50%). Non-host reasons for abstaining included: lack of applicants (n=58, 41%); never considered it (n=40, 28%); insufficient staffing (n= 37, 26%); unsure where to start (n=34, 24%) or find/attract interns (n=34, 24%), and too time-consuming (n=25,18%). Additional reasons for nonparticipation included: lack of space; insufficient intern-suitable work; risk/liability; and agency policy. Both hosting and non-hosting agencies anticipated/realized student-related challenges (e.g., limited motivation, inconsistent availability, inadequate preparation) and agency-based factors (e.g., insufficient time, staffing). Given the ongoing need for suitable gerontology internship sites and the finding that almost half of the non-hosting agencies indicated possible future interest in accepting gerontology interns (n=80, 49%), attention to agency recommendations (e.g., increased faculty communication/feedback, stronger student commitment/preparation, recognition of agency contributions) is warranted. Strategies for addressing identified challenges and implementing requested changes to enhance the community agency experience with gerontology interns are discussed.

